# SARS-CoV-2 testing in patients with low COVID-19 suspicion at admission to a tertiary care hospital, Stockholm, Sweden, March to September 2020

**DOI:** 10.2807/1560-7917.ES.2022.27.7.2100079

**Published:** 2022-02-17

**Authors:** Ana Requena-Méndez, Aikaterini Mougkou, Pontus Hedberg, Suzanne D van der Werff, Hideyuki Tanushi, Olof Hertting, Anna Färnert, Filippa Nyberg, Pontus Naucler, COVID-19-data-review collaborators, COVID-19-data-review collaborators, Isabelle Johansson, Victoria Hovergren, David Björklund, Allan Zhao, Oscar Backrud, Jesper Ahlberg, Emilie Homlkvist, Johanna Lundquist

**Affiliations:** 1 Division of Infectious Diseases, Department of Medicine Solna, Karolinska Institutet, Solna, Sweden; 2 Department of Infectious Diseases, Karolinska University Hospital, Solna, Sweden; 3 Barcelona Institute for Global Health, ISGlobal, Hospital Clinic-Universitat de Barcelona, Barcelona, Spain; 4 Department of Pediatrics, Karolinska University Hospital, Solna, Sweden; 5 Department of Data Processing and Analysis, Karolinska University Hospital, Solna, Sweden; 6 Division of Dermatology and Venereology, Department of Medicine Solna, Karolinska Institutet, Solna Sweden; 7 The COVID-19-data-review collaborators are listed under Investigators

**Keywords:** SARS-CoV-2, coronavirus, COVID-19, screening, testing, low suspicion, hospital admission

## Abstract

**Background:**

Universal SARS-CoV-2 testing at hospital admission has been proposed to prevent nosocomial transmission.

**Aim:**

To investigate SARS-CoV-2 positivity in patients tested with low clinical COVID-19 suspicion at hospital admission.

**Methods:**

We characterised a retrospective cohort of patients admitted to Karolinska University Hospital tested for SARS-CoV-2 by PCR from March to September 2020, supplemented with an in-depth chart review (16 March–12 April). We compared positivity rates in patients with and without clinical COVID-19 suspicion with Spearman’s rank correlation coefficient. We used multivariable logistic regression to identify factors associated with test positivity.

**Results:**

From March to September 2020, 66.9% (24,245/36,249) admitted patient episodes were tested; of those, 61.2% (14,830/24,245) showed no clinical COVID-19 suspicion, and the positivity rate was 3.2% (469/14,830). There was a strong correlation of SARS-CoV-2 positivity in patients with low vs high COVID-19 suspicion (rho = 0.92; p < 0.001).

From 16 March to 12 April, the positivity rate was 3.9% (58/1,482) in individuals with low COVID-19 suspicion, and 3.1% (35/1,114) in asymptomatic patients. Rates were higher in women (5.0%; 45/893) vs men (2.0%; 12/589; p = 0.003), but not significantly different if pregnant women were excluded (3.7% (21/566) vs 2.2% (12/589); p = 0.09). Factors associated with SARS-CoV-2 positivity were testing of pregnant women before delivery (odds ratio (OR): 2.6; 95% confidence interval (CI): 1.3–5.4) and isolated symptoms in adults (OR: 3.3; 95% CI: 1.8–6.3).

**Conclusions:**

This study shows a relatively high SARS-CoV-2 positivity rate in patients with low COVID-19 suspicion upon hospital admission. Universal SARS-CoV-2 testing of pregnant women before delivery should be considered.

## Introduction

Since the first cases of coronavirus disease 2019 (COVID-19) were identified in Wuhan, China, in December 2019, the global pandemic caused by severe acute respiratory syndrome coronavirus-2 (SARS-CoV-2) has led, as at January 2022, to more than 5,600,000 deaths [1]. Although the World Health Organization advocated widespread SARS-CoV-2 testing [2], national capacities for implementing this strategy have diverged considerably because of inadequate testing capacity [3,4].

Hospital screening at admission was implemented from the first weeks of the pandemic to further limit SARS-CoV-2 spread to inpatients and frontline healthcare workers [5]. Symptom-based testing for COVID-19 has shown to be specific but not sensitive, since absence of clinical symptoms does not rule out infections [5]. Furthermore, during the early pandemic, findings emerged suggesting that the virus may spread from asymptomatic or pre-symptomatic individuals [6,7]. SARS-CoV-2 testing approaches focussed solely on the presence of symptoms are therefore unlikely to be adequate to prevent nosocomial spread if there is a sustained community transmission risk [5]. Some hospitals imposed screening to prevent SARS-CoV-2 transmission to operating theatres, oncology units, and labour and delivery wards [8-10], whereas in other hospital units, testing was only performed in symptomatic patients [11].

A report published in 2020 by the European Centre for Disease Prevention and Control (ECDC) on screening strategies recommends that all patients should be tested for SARS-CoV-2 on admission [12]. Whether the use of universal screening on admission is an efficient way of resource allocation to minimise the risk of nosocomial transmission of the virus is depending on the circulation of newly infected individuals in the community what differ among areas and over time [13].

By 15 January 2022, 2,015,276 (19.5%) individuals in the Swedish population have tested positive for SARS-CoV-2 and 0.8% of these died [14]. Stockholm has been one of the most affected regions, with 472,169 (20.1%) confirmed COVID-19 cases and 4,700 deaths (1.0% of positive cases), although only hospital admissions were tested for SARS-CoV-2 in the spring of 2020, resulting in an underestimated number of cases and an overestimated mortality rate [15]. From 25 March 2020 onward, several clinics at Karolinska University Hospital in Stockholm recommended routine SARS-CoV-2 testing to patients admitted to the hospital. This included testing before certain procedures, e.g. before delivery, immunosuppressive treatment, surgery, and other invasive procedures such as endoscopy or bronchoscopy as well as all paediatric admissions (until September 2020), irrespective of whether patients were having a low or high COVID-19 clinical suspicion. 

The primary aim of this study was to assess the proportion of positive SARS-CoV-2 cases identified during healthcare episodes with a low clinical suspicion of COVID-19 tested at hospital admission to Karolinska University Hospital, and to further investigate factors associated with SARS-CoV-2-positive test results among these patients. The secondary aim was to investigate the value of SARS-CoV-2 testing in relation to the background hospitalisation rate because of COVID-19 in Region Stockholm.

## Methods

### Study design and setting

We conducted a retrospective cohort study of all admitted patients, including adults and children, tested for SARS-CoV-2 from 16 March to 27 September 2020 at Karolinska University Hospital (which has two locations in Huddinge and Solna), Stockholm, Sweden. This is a tertiary care academic hospital with 1,100 beds divided between two sites, which serves a population of 2.3 million inhabitants, i.e. the entire population of Region Stockholm. The hospital in Solna is a modern facility that opened in 2017 where most beds are set in private rooms, whereas the hospital in Huddinge includes beds that are located in shared rooms.

### Data sources

We used two sources of healthcare data for this study. First, we used a pseudo-anonymised research database that contains demographic, clinical and microbiological information on all inpatients at Karolinska University Hospital to examine those tested for SARS-CoV-2 at admission from 16 March to 27 September 2020. The research dataset from the entire study period only included structured variables.

Second, to have more information on the reasons for testing and clinical symptoms, we performed an in-depth manual medical chart review of all admitted patients who received a SARS-CoV-2 test during the peak of virus transmission between 16 March to 12 April 2020, using the hospital electronic health record system, as part of a quality assurance project. For the in-depth medical chart review, medical records of healthcare episodes with at least one PCR test for SARS-CoV-2 were manually reviewed; we registered (i) reasons for testing as noted by health professionals, (ii) demographic characteristics, (iii) information on medical history and (iv) clinical and laboratory data when available. All patients in the chart review are included in the research database; however, because personal identification numbers were not available in the research database, it was not possible to directly link the two data sources. 

### SARS-CoV-2 testing

From 1 March 2020, all patients with COVID-19-related symptoms were recommended to be tested for SARS-CoV-2 infection. From 25 March 2020 onward, several clinics at Karolinska University Hospital recommended routine SARS-CoV-2 testing to patients admitted to the hospital (before delivery, immunosuppressive treatment, surgery, and other invasive procedures such as endoscopy or bronchoscopy as well as all paediatric admissions), restricted to tests performed from 24 h before until 48 h after hospital admission. Children were considered those aged < 18 years. For both datasets, PCR tests were performed at the Karolinska University Laboratory according to routine quality assured procedures for SARS-CoV-2 RNA detection (Supplementary File S1). 

A ‘healthcare episode’ was defined as the time from patient admission until discharge from the hospital. New hospital admissions occurring within 12 h following discharge were considered as part of the same healthcare episode. 

### Case definitions

The case definition for patients considered to have a high or low clinical suspicion of COVID-19 varied depending on which dataset was used, i.e. the research dataset (entire study period) vs in-depth medical chart review (16 March to 27 September 2020). 

For the research data set, patients admitted with fever and dyspnoea were considered to have high clinical suspicion of COVID-19. Therefore, we considered patients without fever and dyspnoea on admission as a proxy for low COVID-19 suspicion, i.e. temperature < 38°C AND oxygen saturation ≥ 95% AND respiratory rate depending on age (≤ 60 breaths/min for < 12 months, ≤ 40 for 1–3 years, ≤ 34 for 4–5 years, ≤ 30 for 6–12 years, and ≤ 20 for ≥ 12 years).

For the in-depth manual medical chart review, the reason for testing was defined as ‘high clinical suspicion of COVID-19’ if there were clinically compatible symptoms indicative of SARS-CoV-2 infection including fever AND any respiratory symptoms (cough, dyspnoea, sore throat), and/or if health professionals suspected COVID-19. Healthcare episodes with a high clinical suspicion of COVID-19 together with other high-risk exposure episodes, e.g. transfer from a nursing home stay or from another hospital OR previous hospitalisation, were excluded from further analysis. Testing in healthcare episodes that did not fulfil this definition of high clinical suspicion were regarded as ‘screened with low clinical suspicion of COVID-19’. Yet, since symptoms of COVID-19 can be diffuse, we further assessed these patients according to presence of any symptoms that could be indicative of SARS-CoV-2 infection compared with asymptomatic patients. Individuals were considered asymptomatic if they had no COVID-19-related symptoms including fever, cough, sore throat, fatigue, dyspnoea, myalgia, diarrhoea, abdominal pain, vomiting or headache.

Reasons for testing among those with low COVID-19 suspicion were divided into ‘before surgery’ or any other invasive procedure’, ‘before delivery' and testing at hospital admission when the reason for admission was ‘other than surgery or invasive procedure’.

### Data sources and analysis

The primary outcome was the positivity rate among all individuals with low clinical COVID-19 suspicion. We also examined the positivity rate in asymptomatic patients. Both were evaluated during the 4-week study period beginning on the 16 March 2020. In addition, factors that could be associated with the SARS-CoV-2 infection, i.e. demographics, underlying conditions, symptoms and reasons for testing, were investigated. The testing rate was expressed as the percentage of total SARS-CoV-2 tests conducted per healthcare episode. If there were several healthcare episodes for one patient during the 4-week period, the first episode tested was selected to calculate the positivity rate.

As a secondary analysis, the weekly positivity rate in patients without high-risk symptoms, e.g. fever and dyspnoea, was estimated over the entire study period and compared with patients admitted to the hospital with high clinical suspicion and also with the cumulative COVID-19 hospitalisation rate in Region Stockholm using Spearman’s rank correlation coefficient. The hospitalisation rate was preferred to the incidence at the community level since it reflects better the evolution of the epidemic in the region because of the limited testing capacity outside of hospitals.

Data on sociodemographic level of municipalities were extracted from the Statistical agency of Sweden [16].

Data for the COVID-19 weekly Stockholm hospitalisation rate were facilitated by the Centrum for Epidemiology and Community Medicine (https://ces.sll.se).

### Statistical methods

Summary statistics were presented as proportions for categorical variables and as means with standard deviations (SD) for normally distributed continuous variables or medians with interquartile range (IQR) for skewed continuous variables. Associations were examined with a t-test for normally distributed continuous variables, a Wilcoxon–Mann–Whitney U test for variables not following a normal distribution and a chi-squared test or Fisher’s exact test for categorical variables, as appropriate. The overall positivity rate of SARS-CoV-2 infection was described using 95% confidence intervals (CI). Positivity rate estimates were also obtained for the 4-week period when information on screening or reason for testing was available from the medical records. Outcomes of statistical tests were considered significant when two-sided p < 0.05. A multivariable logistic regression with penalised maximum likelihood estimation was used to identify associations between patient characteristics and a positive test. Variables that were assessed as clinically relevant or those with a significance level of p < 0.1 in the univariate analysis were included for multivariable analysis. Data analyses were performed using STATA (version 16, STATACorp, College Station, Texas, United States).

The study has been reported following the Strengthening the Reporting of Observational Studies in Epidemiology (STROBE) guidelines for reporting observational studies.

### Ethical statement

Ethical approval to use the research database was obtained from the Regional Ethical Review Board in Stockholm (Dnr-2018/1030–31, amendment 2020–01385). The medical chart review was performed as part of the quality control and safety tasks of the hospitals.

## Results

**Figure 1:**
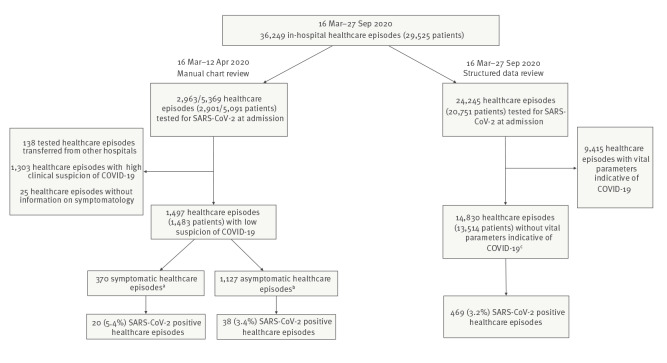
Flowchart of the study population with in-hospital healthcare episodes and SARS-CoV-2 tests, Karolinska University Hospital, Stockholm, Sweden, 16 March–27 September 2020 (n = 36,249 healthcare episodes)

COVID-19: coronavirus disease; SARS-CoV-2: severe acute respiratory syndrome coronavirus 2.

^a^ Low clinical suspicion of COVID-19 with isolated symptoms (fever, cough, fatigue, dyspnoea, myalgia, diarrhoea, abdominal pain, vomiting or headache).

^b^ Low clinical suspicion of COVID-19 without fever, cough, fatigue, dyspnoea, myalgia, diarrhoea, abdominal pain, vomiting and headache.

^c^ Health care episodes with body temperature <38° C, saturation >95% and age-dependent increased respiratory rate (≤60 when <12 months, ≤40 when 1-3 years, ≤34 when 4-5 years, ≤30 when 6-12 years, ≤20 when >12 years).

In total, 36,249 in-hospital healthcare episodes were reported from 16 March to 27 September 2020 and, of these, 24,245 (66.9%) were tested for SARS-CoV-2 by PCR at admission (98.5% nasopharyngeal swabs and 1.5% throat swabs). Among these, 38.8% (9,415/24,245) of SARS-CoV-2-tested healthcare episodes presented with high clinical suspicion, whereas 61.2% (14,830/24,245) of SARS-CoV-2-tested healthcare episodes included patients that presented with low clinical suspicion upon hospital admission. Of these healthcare episodes, 3.2% (469/14,830) were positive for SARS-CoV-2 (Figure 1).

Between 16 March to 12 April 2020, the period targeted for medical chart review, there were 5,369 healthcare episodes in 5,091 individuals; SARS-CoV-2 tests were conducted in 55.2% (2,963/5,369) of healthcare episodes, and 57.0% (2,901/5,091) of these patients. After exclusion of patients transferred from other hospitals (n = 138), patients with a high COVID-19 suspicion (n = 1,303), and healthcare episodes without information about the symptoms (n = 25), 1,497 episodes (from 1,483 patients) were tested and defined as belonging to care episodes with low COVID-19 suspicion (Figure 1).

In total, 39.2% (259/661) of healthcare episodes in children less than 18 years of age were tested with low COVID-19 suspicion, compared with 38.9% (1,237/3,183) of healthcare episodes in adults (p = 0.9; n = 59 missing values on age, of which one was positive and 58 were negative). The testing rate varied across hospital units, which was higher in the maternity units (63.7%; 411/645) compared with the adult surgical or medical emergency units (42.1%; 215/511), paediatric units (38.9%; 231/594), surgical units (38.0%; 222/585), and the medical units (24.4%; 291/1,195; p < 0.001). On 25 March, several units in the hospital implemented routine SARS-CoV-2 testing at admission. The COVID-19 low suspicion testing rate was 19.0% (322/1,694) before 25 March, compared with 53.2% (1,175/2,209) after introducing routine testing (p < 0.001). In the maternity units, the testing rate changed from 11.9% (28/235) before 25 March to 93.4% (383/410) afterwards, and was the unit that had the greatest increase in routine testing rate (p < 0.001).

When the reason for testing was investigated among the 1,497 healthcare episodes tested for SARS-CoV-2 with low suspicion, 21.9% (328/1,497) of the episodes were tested before delivery, 21.7% were (325/1,497) before surgery or other invasive procedure, and 56.4% (844/1,497) were because of any other reason for hospital admission.

**Figure 2:**
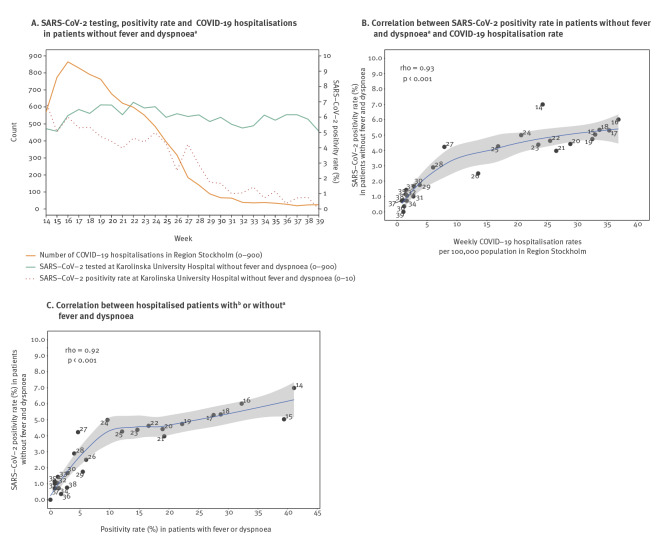
SARS-CoV-2 positivity rate in patients with low clinical suspicion of COVID-19, Karolinska University Hospital, Stockholm, Sweden

COVID-19: coronavirus disease; KUH: Karolinska University Hospital; SARS-CoV-2: severe acute respiratory syndrome coronavirus 2.

^a^ Presenting on admission with a body temperature of < 38 °C, AND an oxygen saturation of > 95%, AND a respiratory rate depending on age as follows: < 60 for < 12 months, < 40 for 1–3 years, < 34 for 4–5 years, < 30 for 6–12 years, and < 20 for > 12 years on admission.

^b^ Presenting on admission with an increased body temperature OR decreased oxygen saturation OR increased respiratory rate according to footnote a.

When comparing the cumulative COVID-19 hospitalisation rate in Region Stockholm per week with the SARS-CoV-2 positivity rate in patients without fever or dyspnoea admitted from March 25 (implementation of more extensive testing at the hospital) until September 27, there was a strong correlation (rho = 0.93; p < 0.001, Spearman’s rank correlation coefficient) (Figure 2A and 2B).

Comparisons of SARS-CoV-2 positivity rate in patients without vital parameters indicative of COVID-19 at Karolinska University Hospital and Region Stockholm COVID-19 hospitalisations and hospitalisation rates per 100,000 population by week. Panel A shows the number of SARS-CoV-2 tests (green line) and the corresponding positivity rate (red dashed line) in patients without fever and dyspnoea at Karolinska University Hospital in relation to the number of COVID-19 hospitalisations in Region Stockholm (orange line). Panel B shows the correlation between the weekly Region Stockholm COVID-19 hospitalisation rate per 100,000 population (black solid circles represent week numbers in 2020) and the weekly positivity rate in patients without fever and dyspnoea at Karolinska University Hospital. Panel C shows the correlation between the weekly positivity rate in patients with (x-axis) or without (y-axis) fever and dyspnoea.

For Panels B and C, rho represents the Spearman’s rank correlation coefficient. The blue line represents a fitted general additive model and the grey area represents the 95% confidence interval of the model.

In analyses restricted to maternity wards, the overall positivity rate was 3.7% (149/4,031) from 25 March until 27 September, and the positivity rate was also correlated with the Region Stockholm cumulative COVID-19 hospitalisation rate over time (rho = 0.75; p < 0.001, Spearman’s rank correlation coefficient) (Supplementary File S2).

When comparing the weekly SARS-CoV-2 positivity rate in patients admitted with and without fever or dyspnoea at Karolinska University Hospital, there was also a strong correlation with positivity rates in those tested with high or low clinical suspicion of COVID-19 (rho = 0.92; p < 0.001, Spearman’s rank correlation coefficient) (Figure 2C).

### Factors associated with SARS-CoV-2 positivity rate among patients with a low clinical COVID-19 suspicion

The main characteristics of the 1,482 patients (one patient with missing age was excluded from the analysis) with the first healthcare episodes used for each patient tested with low suspicion of COVID-19 are presented by reason for being tested and by age group (adults vs children) (Table 1).

**Table 1:** General characteristics and SARS-CoV-2 positivity rate of patients tested with a low clinical suspicion of COVID-19, Karolinska University Hospital, Stockholm, Sweden, March 16–April 12 (n = 1,482)

Characteristics	Hospital admission (not surgical or delivery) (n = 831)^a^				Before surgical or invasive procedure (n = 324)				Before delivery (n = 327)		Total (n = 1,482)^a^			
	Adults (n = 647)		Children (n = 184)		Adults (n = 249)		Children (n = 75)		Adults (n = 327)		Adults (n = 1,223)		Children (n = 259)	
	n	%	n	%	n	%	n	%	n	%	n	%	n	%
Sex														
Female	336	51.9	84	45.7	121	48.6	25	33.3	327	100	784	64.1	109	42.1
Male	311	48.1	100	54.3	128	51.4	50	66.7	0	0	439	35.9	150	57.9
Age (years)														
Median (IQR)^a^	59.0 (42.3–71.3)		4.6 (0.1–11.8)		66.4 (45.1–78.5)		6.7 (2.6–12.3)		31.7 (28.1–35.2)		49.4 (33.0–68.3)		5.6 (0.4–12.3)	
Comorbidities														
Hypertension	221/645	34.3	0	0	99	39.8	0	0	6	1.8	326	26.7	0	0
Cardiovascular diseases	216/645	33.5	1	0.5	93	37.3	3	4.0	4	1.2	316	25.6	4	1.5
Diabetes mellitus	88/645	13.6	3	1.6	25	10.0	0	0	4	1.2	117	9.6	3	1.2
Chronic respiratory diseases	77/646	11.9	6	3.3	21	8.4	3	4.0	1	0.3	99	8.1	9	3.5
Chronic hepatic diseases	34/644	5.3	0	0	11	4.4	1	1.3	1	0.3	46	3.8	1	0.4
Chronic renal diseases	59/643	9.2	5	2.7	23	9.2	3	4.0	0	0	82	6.7	8	3.1
Cancer	184/645	28.5	17/182	9.3	79	31.7	8	10.7	0	0	263	21.5	25	9.7
Transplant	34/644	5.3	1	0.5	3	1.2	3/74	4.1	1	0.3	38	3.1	5	1.9
Autoimmune diseases	46/645	7.1	1	0.5	24/247	9.7	1/74	1.4	1	0.3	71	5.8	2	0.8
Current immunosuppression	89/645	13.8	10	5.4	18	7.2	11/74	14.9	2	0.6	109	8.9	21	8.1
Neuromuscular disorders	13/644	2.0	3	1.6	6	2.4	1	1.3	0	0	19	1.6	4	1.5
SARS-CoV-2 infection														
Patients with low COVID-19 clinical suspicion	20/647	3.1	5/184	2.7	6/249	2.4	2/75	2.7	24/327	7.3	50/1,223	4.1	7/259	2.7
Asymptomatic COVID-19 patients	8/413	1.9	2/104	1.9	3/206	1.5	2/69	2.9	20/322	6.2	31/941	3.3	4/173	2.3
Municipality by socioeconomic index^b^														
Level	n = 520		n = 153		n = 192		n = 53		n = 296		n = 1,008		n = 206	
Low	72	13.8	19	12.4	26	13.5	6	11.3	37	12.5	135	13.4	25	12.1
Medium	174	33.5	67	43.8	69	35.9	24	45.3	114	38.5	357	35.4	91	44.2
High	274	52.7	67	43.8	97	53.5	23	43.4	145	49.0	516	49.9	90	43.7

COVID-19: coronavirus disease; IQR: interquartile range; SARS-CoV-2: severe acute respiratory syndrome coronavirus 2. Low clinical suspicion: COVID-19 cases without clinically compatible symptoms indicative of SARS-CoV-2 infection including fever AND any respiratory symptoms (cough, dyspnoea, sore throat), and if health professionals were not registering the clinical suspicion of COVID-19.

^a^ One individual has missing data on age.

^b^ Data on municipality was unknown for 215 individuals.

Number of observations is n if not otherwise specified. Adults are considered those aged ≥ 18 years and children as those aged < 18 years.

Among the 1,482 patients with low COVID-19 suspicion from 16 March to 12 April, the SARS-CoV-2 positivity rate was 3.8% (57/1,482), and was 6.0% (22/368) in symptomatic patients compared with 3.1% (35/1,114; p = 0.014) in asymptomatic patients. The positivity rate in patients with low COVID-19 suspicion varied across the weeks from 1.7% (2/117) in the third week of March to 4.5% (22/488) in the first week of April. In patients with low COVID-19 suspicion, women had a higher positivity rate (5.0%; 45/893) compared with men (2.0%; 12/589; p < 0.003), but when we exclude the group of pregnant women tested before delivery from the analysis, there was no difference in the positivity rate by sex (3.7%; 21/566 vs 2.0%; 12/589; p = 0.13). Adults had a SARS-CoV-2 positivity rate of 4.1% (50/1,223), and children of 2.7% (7/259; p = 0.3), with a median age among SARS-CoV-2-positive adults of 49.4 years (IQR: 33.0–68.3) and 5.6 years (IQR: 0.4–12.3) among SARS-CoV-2-positive children. The age group 18–45 years had a higher positivity rate (5.6%; 32/570) compared with children (2.7%; 7/259), and compared with adults over 45 years of age (2.8%; 18/653; p = 0.02), although there was no significant difference with the group aged 18–45 years if the pregnant women tested before delivery were excluded (3.3%; 8/243; p = 0.9).

The positivity rate among patients with low clinical suspicion of COVID-19 was highest among women before delivery (7.3%; 24/327), followed by those admitted to the hospital for reasons other than surgery or delivery (3.0%; 25/831), and before surgery or other invasive procedure (2.5%; 8/324) (Table 1). Among asymptomatic patients, the equivalent rates were 6.2% (20/322), 1.9% (10/517) and 1.8% (5/275) for those groups, respectively (p = 0.001).

### Characteristics associated with a SARS-CoV-2 infection in patients with low clinical suspicion of COVID-19

Because of the low number of SARS-CoV-2 positive patients among children with low suspicion (n = 7; 2.7%), separate analyses were performed, stratified for adults and children; analysis with the data for children are presented in Supplementary File S3.

In the adult study population, male sex was associated with a lower PCR positivity rate of SARS-CoV-2 after adjusting for age (odds ratio (OR): 0.4; 95% CI: 0.2–0.8; p = 0.02). Having a neoplasia was negatively but not significantly associated with SARS-CoV-2 positivity (8%; 4/50 vs 22.1%; 259/1,171 (OR: 0.4; 95% CI: 0.1–1.2; p = 0.09)), after adjusting by age and sex. In total, 38.0% (19/50) patients with a positive SARS-CoV-2 PCR test had at least one symptom associated with COVID-19 compared with 263/1,173 (22.4%) for patients with a negative test (OR: 3.3; 95% CI: 1.8–6.3; p < 0.001), despite being regarded as having low suspicion for COVID-19 according to the medical notes. Five out of 50 (10.0%) individuals with a positive SARS-CoV-2 test were reporting fever without any other respiratory symptoms (OR: 2.8; 95% CI: 1.1–7.6; p = 0.039). In addition, 8% (4/50) were reporting cough without fever associated (OR: 3.8; 95% CI: 1.2–11.5; p = 0.019). Furthermore, diarrhoea (3/50; 6.0%) and myalgia (2/50; 4.0%) were also associated with SARS-CoV-2 positivity after adjusting for age and sex (OR: 4.3; 95% CI: 1.2–15.4; p = 0.026 and OR: 22.3; 95% CI: 4.01–123.7; p < 0.001, respectively) (Table 2). A sensitivity analysis was performed restricting the analysis to those tests conducted at hospital admission after the implementation of the routine testing recommendation at hospital admission (25 March) without any substantial changes in the associations found (Supplementary File S4).

**Table 2:** Characteristics associated with SARS-CoV-2 infection in adult patients with low COVID-19 suspicion, Karolinska University Hospital, Stockholm, Sweden, 16 March–12 April 2020 (n = 1,223)

Characteristics	Positive SARS-CoV-2 test (n = 50)^a^		Negative SARS-CoV-2 test (n = 1,173)^a^		OR	95% CI^b^	p value
	n	%	n	%			
Sex							
Female	42	84.0	742	63.3	0.4	0.2–0.8	0.02
Male	8	16.0	431	36.7			
Age (years)							
Median (IQR)	35.6 (32.0–62.8)		50.3 (33.1–69.0)		0.99	0.098–1.01	0.3
Comorbidities							
Hypertension	10	20.0	316/1,171	27.0	1.1	0.5–2.8	0.8
Cardiovascular disorders	6	12.0	307/1,171	26.2	0.5	0.2–1.4	0.2
Diabetes mellitus	5	10.0	112/1,171	9.6	1.6	0.6–4.4	0.3
Chronic respiratory disorder	4	8.0	95/1,172	8.1	1.3	0.4–4.0	0.5
Chronic hepatic disorder	3	6.0	43/1,170	3.7	2.3	0.7–8.0	0.2
Chronic renal failure	1	2.0	81/1,168	6.9	0.4	0.5–2.9	0.3
Cancer	4	8.0	259/1,171	22.1	0.4	0.1–1.2	0.09
Autoimmune disease	5/49	10.2	66/1,170	5.6	2.2	0.8–5.8	0.1
Immunosuppression	2	4.0	107/1,171	9.1	0.5	0.1–2.3	0.4
Transplantation	1	2.0	37/1,170	3.2	0.9	0.1–7.2	1.0
Symptoms							
Fever (without respiratory symptoms)	5	10.0	60	5.1	2.8	1.1–7.6	0.039
Cough (without fever)	4	8.0	37	3.2	3.8	1.2–11.5	0.019
Sore throat (without fever)	0	0	20/1,172	1.7	0.7	0.04–11.7	0.8^c^
Rhinitis (without fever)	0	0	8	0.7	1.6	0.08–27.8	0.8^c^
Dyspnoea (without fever)	3	6.0	44/1,170	3.8	2.4	0.7–8.7	0.17
Fatigue	3	6.0	37/1,171	3.2	3.1	0.9–11.2	0.077
Diarrhoea	3	6.0	26/1,171	2.2	4.3	1.2–15.4	0.026
Myalgia	2	4.0	3	0.3	22.3	4.01–123.7	< 0.001^c^
Headache	0	0	18/1,172	1.5	0.6	0.03–10.6	0.7
Vomiting	1	2.0	37	3.2	0.9	0.1–7.1	0.94
Dysgeusia/dysosmia	2	4.0	1	0.09	28.9	3.7-224.8	0.001^c^
At least one symptom^d^	19	38.0	263	22.4	3.3	(1.8–6.3)	< 0.001
Clinical findings							
Temperature, median °C (IQR)	n = 20		n = 652		1.3	(0.7–2.3)	0.4
	36.8 (36.6–37.5)		36.9 (36.6–37.3)				
O_2_ saturation, % (IQR)	n = 19		n = 632		0.9	(0. 9–1.0)	0.08
	96 (94–98)		97 (95–99)				

CI: confidence interval; IQR: interquartile range; SARS-CoV-2: severe acute respiratory syndrome coronavirus 2.

^a^ Number of observations is n if not otherwise specified.

^b^ Adjusted for sex and age.

^c^ Penalised maximum likelihood logistic regression (Firth model).

^d^ The individual had at least one COVID-19-related symptom, irrespective of which symptom (fever, cough, dyspnoea, nausea, vomiting, diarrhoea, myalgia, headache, fatigue) before the testing procedure.

Adults are considered those aged ≥ 18 years. Data on children (aged < 18 years) are presented in Supplementary File S3.

Fever and diarrhoea were the only symptoms significantly associated with SARS-CoV-2 positivity in children after adjusting for age and sex (Supplementary File S4).

Using a penalised maximum likelihood multivariable logistic regression model that included age, sex, reason for testing and clinical symptoms, the factors associated with SARS-CoV-2 positivity were being tested before delivery (adjusted OR (aOR): 2.6; 95% CI: 1.3–5.4), and having isolated symptoms such as fever (aOR: 2.9; 95% CI 1.2–7.4), cough (aOR 4.0; 95% CI: 1.4–12.0), diarrhoea (aOR: 3.7; 95% CI: 1.1–12.3) and myalgia (aOR: 14.5; 95% CI 2.6–81.2) (Table 3).

**Table 3:** Multivariable logistic regression model of risk factors for SARS-CoV-2 infection in patients with low COVID-19 suspicion, Karolinska University Hospital, Stockholm, Sweden, 16 March–12 April 2020 (n = 1,482)

**Characteristics**	**aOR**	**95% CI**	**p value**
Age	1.0	0.99–1.02	0.7
Sex (male)	0.5	0.3–1.1	0.09
Reason for being tested			
Hospital admission	Reference	NA	NA
Before surgery	1.2	0.5–2.8	0.6
Before delivery	2.6	1.3–5.4	0.008
Comorbidities and symptoms			
Cancer	0.5	0.2–1.4	0.2
Fever (without respiratory symptoms)	2.9	1.2–7.4	0.02
Cough (without fever)	4.0	1.4–12.0	0.012
Diarrhoea	3.7	1.1–12.3	0.03
Myalgia	14.5	2.6–81.2	0.002

CI: confidence interval; COVID-19: coronavirus disease; OR: odds ratio; SARS-CoV-2: severe acute respiratory syndrome coronavirus 2.

Reference category for binary variables (cancer, fever, cough, diarrhoea, and myalgia) was not having any of these conditions. Having dysgeusia/dysosmia was not included in the final model since the number of events were very small. Penalised maximum likelihood logistic regression model adjusted by sex, age, reason for being tested, comorbidity of cancer and having fever, cough, diarrhoea, and myalgia.

## Discussion

Our study reveals a SARS-CoV-2 test positivity rate of almost 4% among patients with low COVID-19 suspicion at hospital admission during a 4-week period (16 March–12 April) in 2020 when SARS-CoV-2 community transmission was widely established in Region Stockholm [15]. During this period, around 75% (1,115/1,482) of the patients tested with low suspicion were asymptomatic for COVID-19 symptoms, whereas 25% (368/1,482) of patients reported some isolated symptoms, which could be associated with COVID-19 but did not trigger an obvious COVID-19 suspicion by health professionals. In addition, we have excluded those patients who were tested because of a high COVID-19 suspicion; this group had a positivity rate around 40% during the 4-week period (data not shown).

Although patients defined in our study as having low COVID-19 suspicion might not be exactly comparable to a systematically screened hospital population, they represent a patient group in which the decision of performing a SARS-CoV-2 test should be taken at admission to the hospital. The two patient populations in our hospital who were screened more systematically, i.e. before surgery or other invasive procedures in adults and before delivery in pregnant women, had a positivity rate of 2.7% and 7.3%, respectively.

The SARS-CoV-2 test positivity rate we observed in asymptomatic patients (˃ 3%) was similar to that reported in another study during their peak of the epidemic during the first pandemic wave [17], although our estimate is higher compared with screening studies reporting hospital positivity rates below 1% which were conducted in areas with low incidence rates [18,19]. In our study, four of every 100 patients with low COVID-19 suspicion (3/100 in asymptomatic patients) tested positive for SARS-CoV-2 with PCR; this suggests that, in a universal SARS-CoV-2 hospital testing scenario with an established community transmission, the numbers needed to test (NNT) to detect one positive patient with low COVID-19 suspicion would be 26.

The positivity rate in low COVID-19 suspicion cases correlated fairly well with the positivity rate in those with a high COVID-19 suspicion in our study, as well as with the hospitalisation rate in Region Stockholm. In this regard, if the accepted NNT to detect one positive patient in low suspicion patients (a proxy for screening) is 100, our data indicates that this would occur when the positivity rate among high-risk patients exceeds 1.8%. If the accepted NNT is 50, the equivalent positivity rate among high-risk patients is 3.8%. The corresponding figures based on the cumulative COVID-19 hospitalisation rate per week were 1.8 and 3.9/100,000 population. Therefore, the positivity rate of patients with COVID-19 symptoms and the cumulative COVID-19 hospitalisation rate per week could be used to estimate the transmission risk at the hospital. In this regard, and as per recommended by ECDC, the testing approach at hospital admission needs to consider the epidemiological situation in the community served by the hospital. Thus, if the SARS-CoV-2 transmission in the community served by the hospital is very low or absent, universal testing should not be implemented [12]. Additional studies should be designed to elucidate and validate the cut-off value observed in our study, to further guide hospital screening implementation.

The overall positivity rate of women was higher compared with men, although there was no difference when pregnant women admitted before delivery were excluded. In this regard, when comparing the reasons for SARS-CoV-2 testing among those with low suspicion, the SARS-CoV-2 test positivity rate in pregnant women presenting for delivery was undoubtedly higher compared with other screening purposes such as hospital admission or testing before surgery or any other invasive procedure. This was particularly manifested in asymptomatic patients, with a positivity rate over 6% observed in asymptomatic pregnant women before delivery. This high positivity rate has also been reported in other studies with ranges varying from 1.4 to 14.5%, depending on the community prevalence of the area [18,20-22]. On one hand, a plausible explanation is that based on the association of COVID-19 with preeclampsia [23]. On the other hand, most pregnant women with preeclampsia may have been referred to the Karolinska University hospital, where the COVID-19 diagnosis was performed, capturing more COVID-19-positive pregnant women and therefore overestimating the positivity rate found in our study.

Among patients with low suspicion of COVID-19, cancer was associated with a lower SARS-CoV-2 positivity rate, around 0.5% in asymptomatic patients. This low rate suggests that patients with this condition may have adjusted their behaviour, e.g. isolated themselves, and avoided exposure to SARS-CoV-2. However, despite the fact that the SARS-CoV-2 prevalence rate in oncological patients is lower compared with patients in other units such as maternity wards [18], the baseline testing for patients with cancer has been undoubtedly proposed in all inpatients admitted to oncology/haematology units and before starting immunosuppressive chemotherapy [24].

Therefore, when implementing a universal screening policy at hospital admission, testing women presenting for delivery should be a core strategy to reduce the risk of SARS-CoV-2 transmission in healthcare facilities [25], particularly since only around 14 pregnant women were needed to test before delivery to detect 1 positive case. However, concerning testing before elective surgery and other invasive procedures or before hospital admission, we report a lower positivity rate, resulting respectively in 40 and 32 NNT to identify one positive SARS-CoV-2 case, which is slightly higher in asymptomatic patients. Thereafter, for the evaluation of the cost-effectiveness of universal screening policies at hospital admission, other aspects should be considered [19,26] such as the community incidence of infection, since an escalating COVID-19 incidence may increase potential benefits of this policy. In a study from Japan in an area with low levels of community SARS-CoV-2 transmission, the positivity rate was 0.03% (only two cases among more than 6,000 patients tested). This suggests that a universal SARS-CoV-2 testing strategy might be labour-intensive and not cost-effective in areas with low infection rates [26]. Also, the strategy will depend on the variations of the community transmission level across regions or municipalities [27] and also over time. We show that the positivity rate remained high the first months of the epidemic and subsequently declined in the following months, although the testing rate remained stable. In this regard, we propose the use of the positivity rate of patients with COVID-19 symptoms who will definitely be tested for estimating the transmission risk at the hospital in asymptomatic patients and to guide the implementation of screening testing. In addition, other potential benefits to consider are the prevention of unnecessary risks of surgery performed on someone with an underlying COVID-19 infection [28].

There are also barriers and challenges to the implementation of standardised COVID-19 screening programmes. These include the diagnostic testing capacity of the health services, logistical issues affecting sampling and turnaround times [5], and also the possibility of false-negative results for patients tested during the incubation period, for which a re-testing procedure after 5–7 days has been proposed [29].

We identified clinical predictors for a positive SARS-CoV-2 test in patients with low COVID-19 suspicion. In the multivariable model, isolated symptoms, e.g. self-reported fever (without cough), cough (without fever), diarrhoea and myalgia, were associated with test positivity after adjustment for age, sex, the reason for testing and other clinical parameters. These findings indicate that vigilance for possible SARS-CoV-2 infection should remain high, even in patients with primary suspicion of other diagnosis when there is any symptom indicative of SARS-CoV-2, at least when community spread is high.

The low number of SARS-CoV-2-positive children precluded us from performing separate multivariate analyses in children. However, univariate results are in line with other studies where the symptom-based testing strategy failed to detect up to 45% of children [30] suggesting that a symptom-based testing strategy could lead to a substantial increased risk of intra-hospital transmission.

A strength of the study is that we included complete data from one hospital during a period of almost 7 months with more than 24,000 healthcare episodes reviewed and with an in-depth manual record review of almost 3,000 healthcare episodes. A limitation of the study is the retrospective design based on clinical routine data, and in some cases, the reasons for testing may have been poorly registered by health professionals while the reason for testing with low COVID-19 suspicion may be overestimated. To reduce the risk of misclassification, we excluded patients tested because of high COVID-19 suspicion and patients with more than one symptom suggestive of COVID-19. Our study has limited generalisability to areas with different rates of SARS-CoV-2 infections. Furthermore, we cannot conclude that our findings are representative of all patients diagnosed with COVID-19 since the study only included patients admitted to the hospital.

### Conclusions

This study reports a high SARS-CoV-2 test positivity rate in patients with low COVID-19 suspicion at hospital admission in Stockholm from March to September 2020. Universal testing before delivery and testing in those patients with any isolated symptom despite having low COVID-19 suspicion should be considered among SARS-CoV-2 testing strategies at the hospital level, although the background incidence at the community should be considered. Positivity rates in hospitalised patients with high-suspicion of COVID-19 can guide SARS-CoV-2 screening implementation.

## Supplementary Data

329000 Supplement
