# Supplementary material for: SARS-CoV-2 testing in patients with low COVID-19 suspicion at admission to a tertiary care hospital, Stockholm, Sweden, March to September 2020
Source: Euro Surveill. 2022 Feb 17;27(7):2100079. doi: 10.2807/1560-7917.ES.2022.27.7.2100079 (PMC8855509; doi:10.2807/1560-7917.ES.2022.27.7.2100079)
Supplement: Supplement [file 21-00079_MENDEZ_Supplement.pdf]

SUPPLEMENTARY FILES

Disclaimer:

This supplementary material is hosted by Eurosurveillance as supporting information alongside the article "SARS-Co-V-2 testing in patients with low COVID-19 suspicion at hospital admission in a third-level hospital in Stockholm: March-September 2020", on behalf of the authors, who remain responsible for the accuracy and appropriateness of the content. The same standards for ethics, copyright, attributions, and permissions as for the article apply. Supplements are not edited by Eurosurveillance and the journal is not responsible for the maintenance of any links or email addresses provided therein

Supplementary file 1: PCR Methods

PCR analyses were performed at the Karolinska University Laboratory according to normal routine procedures for SARS-CoV-2 RNA detection. Airway samples were analysed on the cobas® 6800 instrument, a fully automated test including RNA extraction (Roche Molecular Diagnostics, Pleasanton, CA, USA) or an in-house method with RNA-extraction (MagNA Pure 96, Roche Diagnostics) followed by a modified version (unpublished) of the Drosten protocols for PCR analysis.

For the in-house assay, the cut-off is ct 40, for the commercial assays the cut-off accordingly to the manufacturer's instructions. The two PCR methods had similar performance in in-house assessments.

The cobas® target the envelope (E) and Open Reading Frame (ORF) 1 genes. The in-house assay targets the E-gene and the RNA-dependent RNA polymerase (RdRp) gene. Amplification of at least one gene was considered a positive test

For the in-house PCR and the Amplidiag assay, dual positive samples are reported as positive, single positive samples are re-run and reported positive if repeatedly positive.

For the cobas assay, dual positive and single-positive in the ORF1 gene are reported as positive, single positive E gene samples are re-run.

Further information about the PCR tests can be found in the supplementary material of the following reference:

Hagman K, Hedenstierna M, Gille-Johnson P, Hammas B, Grabbe M, Dillner J, et al. Severe Acute Respiratory Syndrome Coronavirus 2 RNA in Serum as Predictor of Severe Outcome in COVID-19: A Retrospective Cohort Study. Clin Infect Dis 2020 Aug 28;ciaa1285; Available at: https://pubmed.ncbi.nlm.nih.gov/32856036/.

Supplementary file 2. Correlation between the hospitalization rate in Region Stockholm and the positivity rate in the maternity wards at Karolinska University Hospitals.

2A. Karolinska University Hospital maternity ward SARS-CoV-2 testing, positivity rate and Region Stockholm COVID-19 hospitalisations in patients without fever and dyspnoea [a]

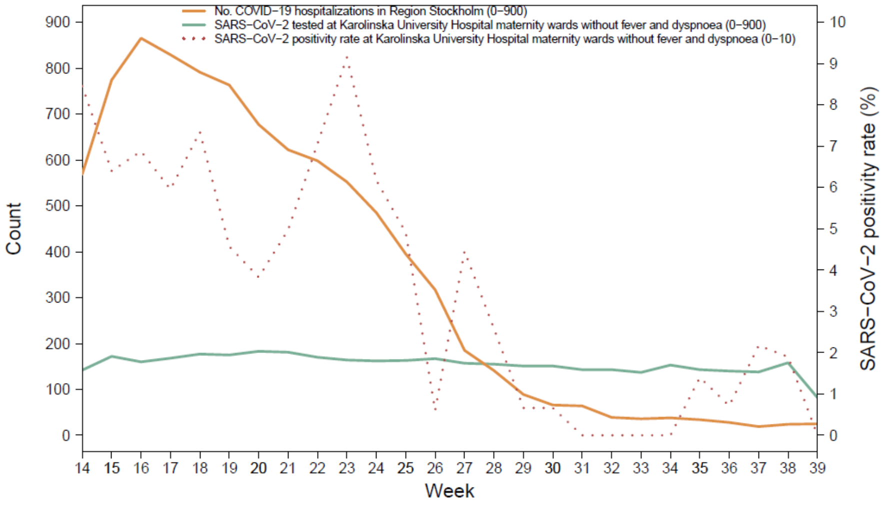

2B. Correlation between SARS-CoV-2 positivity rate in patients without fever and dyspnoea [a] at Karolinska University Hospital maternity wards and Region Stockholm COVID-19 hospitalization rate

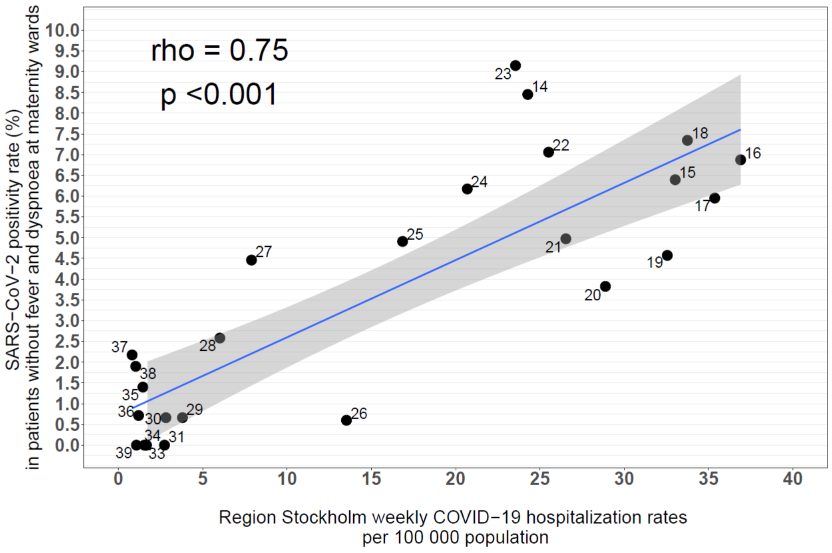

Legend: Comparisons of SARS-CoV-2 positivity rate in patients without vital parameters indicative of COVID-19 at Karolinska University Hospital maternity wards and Region Stockholm COVID-19 hospitalisations and hospitalisation rates per 100 000 population by week. Panel A shows the number of SARS-CoV-2 tests (green line) and the corresponding positivity rate (red dashed line) in patients without fever and dyspnoea at Karolinska University Hospital maternity wards in relation to the number of COVID-19 hospitalisations in Region Stockholm (orange line). Panel B shows the correlation between the weekly Region Stockholm COVID-19 hospitalization rate per 100 000 population (numbered dots) and the weekly positivity rate in patients without fever and dyspnoea at Karolinska University Hospital maternity wards. rho = 0.93 represents the Spearman's rank correlation coefficient. The blue line represents a fitted general additive model and the grey areas represent the 95%CI of the model. [a] Presenting on admission with a body temperature of <38 degrees Celsius, AND an oxygen saturation of >95%, AND a respiratory rate depending on age as follows <60 for <12 months old, <40 for 1-3 year old, <34 for 4-5 year old, <30 for 6-12 year

old, and <20 for >12 year old on admission. [b] Presenting on admission with an increased body temperature OR decreased oxygen saturation OR increased respiratory rate.

Supplementary file 3. Factors associated with SARS-CoV-2 infection among children with low suspicion of SARS-CoV-2 (n=259)

		Positive SARS-CoV-2 test (n=7 if not otherwise specified), n (%)[1]	Negative SARS-CoV-2 test (n=252 if not otherwise specified), n (%)[1]	Odds Ratio (95% CI)[2]	p-value[3]
	Sex (male)	4 (57.1)	146 (57.9)	1.0 (0.2-4.2)	1
	Age, years (Median, IQR)	9.3 (0.07-13.3)	5.5 (0.4-12.1)	1.0 (0.9-1.2)	0.4
**Comorbidities**	Hypertension	0	0	---	---
	Cardiovascular diseases	0	4 (1.6)	3.5 (0.2-74.9)	0.43
	Diabetes mellitus	0	3 (1.2)	4.0 (0.2-85.7)	0.37
	Chronic respiratory disorder	0	9 (3.6)	1.5 (0.8-27.9)	0.79
	Chronic hepatic disorder	0	1 (0.4)	7.9 (0.3-232.6)	0.23
	Chronic renal failure	0	8 (3.2)	1.9 (0.1-34.9)	0.68
	Cancer	0	25/250 (10.0)	0.6 (0.03-10.1)	0.70
	Autoimmune disease	0	2/251 (0.8)	5.2 (0.2-135.9)	0.32
	Immunosuppressant drugs	0	21/251 (8.4)	0.6 (0.03-10.9)	0.72
	Transplantation	0	5/251 (2.0)	2.1 (0.10-45.8)	0.64
**Symptomatology**	Fever without cough	3 (42.9)	36 (14.3)	**5.9 (1.1-30.7)**	**0.036**
	Cough without fever	0	8 (3.2)	1.9 (0.1-35.6)	0.68
	Sore throat	0	2 (0.8)	4.4 (1.2-113.1)	0.38
	Rhinitis	0	6 (2.4)	2.6 (0.1-49.8)	0.53
	Dyspnoea	0	7/251 (2.8)	2.6 (0.1-52.4)	0.53
	Fatigue	1 (14.3)	12/251 (4.8)	5.5 (0.7-41.1)	0.10
	Diarrhea	1 (14.3)	7/251 (2.8)	**7.8 (1.2-53.3)**	**0.04**
	Myalgia	1 (14.3)	0	--	---
	Headache	0	5/251 (2.0)	2.5 (0.1-50.1	0.5
	Vomiting	0	25/249 (10.0)	0.6 (0.03-0.9)	---
	At least one symptom	3 (42.9)	83 (32.9)	1.6 (0.4-7.7)	0.1
**Clinical findings**	Temperature, C median (IQR)	37.3 (36.1-38.6) n=4	37.1 (36.7-37.6) n=194	1.1 (0.4-3.0)	0.8
	O2 Saturation, %, median (IQR)	98 (98-100) n=3	98 (98-100) n=180	1.1 (0.6-2.2)	0.7

1. Presented as number and percentage if not otherwise specified; *2. Adjusted for sex and age; 3. Penalized maximum likelihood logistic regression (Firth model)

Supplementary file 4. Restricted analysis of factors associated with SARS-CoV-2 infection among adults with low suspicion of SARS-CoV-2 after March 25 (Implementation of the screening recommendations)

		Positive SARS-CoV-2 test (n=48[1]), n (%)[2]		Negative SARS-CoV-2 test (n=996[1]), n (%)[2]		Odds Ratio (95% CI)[3]	p-value
		n	%	n	%		
Comorbidities	Sex (male)	7	14.6	334	**33.5**	**0.4 (0.2-0.8)**	**0.02**
	Age (Median, IQR)	35.6 (32.1-63.1)		44.4 (32.2-67.4)		1.0 (.098-1.01)	0.7
	Hypertension	9	18.8	254/994	25.6	0.9 (0.4-2.4)	0.9
	Cardio-vascular disorders	5	10.4	248/994	25.0	0.4 (0.1-1.2)	0.1
	Diabetes mellitus	4	8.3	87/994	8.8	1.4 (0.5-4.2)	0.6
	Chronic respiratory disorder	3	6.3	76/995	7.6	1.0 (0.3-3.4)	1.0
	Chronic hepatic disorder	3	6.3	32/994	3.2	2.9 (0.8-10.4)	0.1
	Chronic renal failure	1	2.1	54/991	5.5	0.5 (0.06-3.6)	0.5
	Cancer	4	8.3	200/994	20.1	0.4 (0.2-1.32)	0.09
	Autoimmune disease	5/47	10.6	54/993	5.4	2.2 (0.8-6.1)	0.1
	Immunosuppression	2	4.2	63/994	6.3	0.8 (0.2-3.4)	0.7
	Transplantation	1	2.1	22/993	2.2	1.3 (0.2-10.3)	0.8
Symptomatology	Fever without respiratory symptoms	5	10.4	41/996	**4.1**	**3.4 (1.2-9.2)**	**0.014**
	Cough (without fever)	4	8.3	27/996	**2.7**	**4.2 (1.3-12.9)**	**0.019**
	Sore throat (without fever)	0	0	13/993	1.3	0.9 (0.05-14.8)	0.9[4]
	Rhinitis (without fever)	0	0	6/996	0.6	1.6 (0.09-28.7)	0.8[4]
	Dyspnoea (without fever)	2	4.2	35/993	3.5	1.5 (0.3-6.7)	0.6
	Fatigue	3	6.0	37/1171	3.2	**3.9 (1.1-14.5)**	**0.039**
	Diarrhea	3	**6.0**	**26/1171**	**2.2**	**4.8 (1.3-17.6)**	**0.019**
	Myalgia	2	**4.2**	**3/996**	**0.2**	**29.9 (4.6-195.1)**	**<0.001[4]**
	Headache	0	0	13/995	1.3	0.7 (0.04-12.7)	0.8
	Vomiting	1	2.1	32/996	3.2	0.9 (0.1-6.7)	0.9
	Dysgeusia/dysosmia	2	4.2	1/996	0.1	27.3 (3.5-212.0)	0.002[4]
	At least one symptom[5]	18	**37.5**	**207/996**	**20.8**	**3.4 (1.7-6.5)**	**<0.001**
Clinical findings	Temperature (median, IQR) °C	36.7 (36.6-37.5)		36.9 (36.6-37.3) n=514		1.4 (0.7-2.8)	0.3
	O2 Saturation %	96.5 (95-98) n=19		98 (96-99) n=640		0.9 (0.8-1.0)	0.2

1.Number of observations is n if not otherwise specified; 2. Presented as number and percentage if not otherwise specified; 3. Adjusted for sex and age; 4. Penalized maximum likelihood logistic regression (Firth model); 5 At least one symptom, means that individual had any COVID-19 related symptom, irrespective of which symptom, before the testing procedure.
